# Psychometric Properties of the Stress and Anxiety to Viral Epidemics-6 Items (SAVE-6) Scale for High School Students During the COVID-19 Pandemic

**DOI:** 10.3389/fpsyt.2022.815339

**Published:** 2022-05-04

**Authors:** Taeyeop Lee, Oli Ahmed, Ömer Faruk Akça, Seockhoon Chung

**Affiliations:** ^1^Department of Psychiatry, Asan Medical Center, University of Ulsan College of Medicine, Seoul, South Korea; ^2^Department of Psychology, University of Chittagong, Chattogram, Bangladesh; ^3^National Centre for Epidemiology and Population Health, Australian National University, Canberra, ACT, Australia; ^4^Department of Child and Adolescent Psychiatry, Meram School of Medicine, Necmettin Erbakan University, Konya, Turkey

**Keywords:** COVID-19, epidemics, anxiety, stress, psychological

## Abstract

**Objectives:**

Owing to the COVID-19 pandemic, high school students have experienced a sudden change of school environment, which may result in difficulties related to mental health. The aim of this study is to estimate the reliability and validity of the Stress and Anxiety to Viral Epidemics-6 Items (SAVE-6) scale among high school students.

**Methods:**

A cross-sectional online survey was conducted among 300 high school students. The following scales were administered: the SAVE-6, Generalized Anxiety Disorder-7 Items (GAD-7), and Patient Health Questionnaire-9 Items (PHQ-9). Exploratory factor analysis (EFA) and confirmatory factor analysis (CFA) were conducted, and the psychometric properties of the SAVE-6 were assessed.

**Results:**

The results of the CFA indicated good model fit for the SAVE-6 scale among high school students (χ2/df = 0.485, CFI = 1.000, TLI = 1.010, RMSEA < 0.001, and SRMR = 0.029). In addition, the SAVE-6 scale demonstrated good reliability (Cronbach’s alpha = 0.844, McDonald’s Omega = 0.848, and split-half reliability = 0.883). The appropriate cut-off score for the SAVE-6 scale was estimated as ≥15, which corresponds to a mild level of anxiety as assessed by the GAD-7 (≥5).

**Conclusion:**

The SAVE-6 scale was found to be reliable and valid, and can be used as a tool to assess the stress and anxiety of high school students during the COVID-19 pandemic.

## Introduction

The novel coronavirus (COVID-19) is an infectious disease that was first reported in Wuhan, Hubei Province, China. Since the first report in December 2019, COVID-19 rapidly spread to neighboring countries, and in March 2020, the World Health Organization (WHO) declared a consequent global pandemic.

In South Korea, the COVID-19 outbreak in 2020 occurred just before the beginning of the new school year, which is typically scheduled for March 2. The Ministry of Education postponed the commencement of the new school year for 5 weeks, and recommended online self-learning materials in the interim (1). As the pandemic prolonged, official preparations were made for online schooling, which began for the first time in Korean education history on April 9 (2). When school reopened in mid-May, several changes occurred in the classrooms. The students were regularly subject to health checks for the presence of fever or respiratory symptoms; they were required to adhere to social distancing guidelines and avoid close contact with peers. In addition, schools closed and reopened irregularly, according to changes in the social distancing guidelines announced by the Korean government. When schools were closed, online classes were conducted, but challenges, such as issues regarding technology use and difficulties in communication, were reported (3).

Based on the sudden change in the school environment, high school students, who spend a longer time at school, reported difficulties related to mental health (1). Attending school irregularly results in a loss of routine as well as increased feelings of loneliness and isolation. Developmental changes render high school adolescents highly dependent on peer groups, and friends become the primary source of social interaction. The COVID-19 pandemic has forced high school students to be socially isolated, increasing the likelihood of mental health problems including anxiety and stress (4). As an attempt to be socially connected during the pandemic, high school students may seek interaction on social media. However, social media use during the pandemic has been linked to negative mental health outcomes (5). Increased social media use and attending online classes result in excessive screen time, which may increase the risk of insomnia and anxiety (6). Thus, high school students are vulnerable to stress and anxiety during the pandemic, and an efficient screening tool is needed to identify and aid this at-risk group.

The Stress and Anxiety to Viral Epidemics-6 Items (SAVE-6) was developed as a self-rating scale for measuring one’s anxiety response to the COVID-19 pandemic (7). It was based on the original SAVE-9, which was developed to measure healthcare workers’ work-related stress and anxiety response to the viral epidemic (8). The reliability and validity of factor I of the SAVE-9 scale (namely, SAVE-6) were tested among the general population in South Korea (7), Lebanon (9), and the United States (10), as well as among special populations such as medical students (11), public workers (12), and cancer patients (13). Thus far, all participants in such validation studies have been ≥18 years old. Considering that scales developed in one population should be re-evaluated when applied to another population with different characteristics (14), it is necessary to assess the psychometric properties of the SAVE-6 among high school students. In this study, we aimed to explore the reliability and validity of the SAVE-6 scale among a sample of high school students in South Korea and to examine its applicability in measuring students’ anxiety response to the COVID-19 pandemic.

## Method

### Participants and Procedure

This online survey was conducted in South Korea from October 18–24, 2021. Until this period, 40,599,114 (78.4%) individuals among the general population in Korea were vaccinated at least once, 33,966,716 (65.6%) received both shots of the vaccination, and 11,022 had received the booster shot (15). All 300 high school students voluntarily participated in this survey through a professional survey company, EMBRAIN^1^. The survey was conducted anonymously, and no personal identifiable information was collected. The study protocol was approved by the Institutional Review Board of the Asan Medical Center (2021-1361), which waived the requirement to obtain written informed consent.

The survey form included questions on participants’ age, sex, grade, type of school, living area, and responses to questions on COVID-19 including: “Did you experience being quarantined due to infection with COVID-19?”, “Did you experience being infected with COVID-19?”, “Did you get vaccinated?”, and “Do you want to get vaccinated, if the vaccine is available?” Participants’ past psychiatric history was assessed through the question: “Have you experienced or been treated for depression, anxiety, or insomnia?” Furthermore, current psychiatric symptoms were assessed by the question: “Presently, do you think you are depressed or anxious, or do you need help for your mood state?” The survey form was developed in Korean and followed the Checklist for Reporting Results of Internet e-Surveys (CHERRIES) guidelines (16). After the e-survey was developed by the survey company, the usability and technical functionality was tested by an author of this study (TL) before its implementation.

The sample size was determined as 300 high school students, as a sample of 200–300 participants is considered appropriate for factor analysis in the development of a scale (17, 18). Furthermore, the sample size estimation was 30 participants per cell, indicative of a subsample (19). We allocated 50 samples each to six cells: biological sex (boy and girl) and grade (1st, 2nd, or 3rd). The company sent emails to 4,000 high school student panelists for study enrollment; of these, 1,183 panelists accessed this survey, and 354 completed it. Consent was obtained from the parents who agreed that their children could participate in the survey. Following these steps, we collected 300 participants’ responses, representing 0.00023% of all registered high school students (1,299,965) in South Korea (20). The collected data were delivered to investigators, after the survey company excluded all identifiable private information.

## Measures

### Stress and Anxiety to Viral Epidemics-6 Items (SAVE-6)

The SAVE-6 scale (7) was developed from factor I of the SAVE-9 scale, which was originally developed to measure healthcare workers’ work-related stress and anxiety response to the viral epidemic^2^. Specifically, the SAVE-6 can measure one’s anxiety response to a viral epidemic. It consists of six items rated on a five-point Likert scale ranging from 0 (never) to 4 (always). The total score of the SAVE-6, which ranges from 0 to 24, reflects the levels of anxiety response to a viral epidemic. A higher score indicates a severe degree of anxiety. In this study, we applied the original SAVE-6, which was developed in Korean, to high school students without any modification.

### Generalized Anxiety Disorder-7 Items

The Generalized Anxiety Disorder-7 Items (GAD-7) is a rating scale that can measure the severity of general anxiety (21). It consists of seven items rated on a four-point Likert scale ranging from 0 (not at all) to 3 (nearly every day). The total GAD-7 score ranges from 0 to 21, and a high score indicates severe general anxiety. In this study, we defined clinical anxiety as GAD-7 ≥ 10. We applied the Korean version of GAD-7^3^. Cronbach’s alpha for this sample was 0.922.

### Patient Health Questionnaire-9 Items

The Patient Health Questionnaire-9 Items (PHQ-9) is a rating scale that can measure the severity of depression (23). It consists of nine items rated on a four-point Likert scale ranging from 0 (not at all) to 3 (nearly every day). The total PHQ-9 score ranges from 0 to 27, and a high score indicates severe depression. In this study, we defined clinical depression as a PHQ-9 score ≥ 10. We applied the Korean version of PHQ-9 (see footenote 3). Cronbach’s alpha for this sample was 0.910.

## Statistical Analysis

First, we conducted exploratory factor analysis (EFA) and confirmatory factor analysis (CFA) to explore the factor structure of the SAVE-6 scale among high school students. The normality assumption was checked based on the skewness and kurtosis (acceptable range = ± 2) (24) of all six items. In the EFA, data suitability and sampling adequacy were assessed through the Kaiser-Meyer-Olkin (KMO) value and Bartlett’s test of sphericity. In the CFA, model fit was assessed through the χ2/df ratio, comparative fit index (CFI), Tucker-Lewis index (TLI), root-mean-square-error of approximation (RMSEA), and standardized root-mean-square residual (SRMR) values (25, 26). A multi-group CFA was run to assess whether the SAVE-6 can measure the anxiety response of high school students in a similar manner across the variables of sex, grade, and depression (PHQ-9 ≥ 10). Next, the psychometric properties of this scale were assessed through the modern test theory approach (graded response model [GRM]). The GRM provides two types of statistics: slope parameter and threshold parameter. Before running the GRM, assumptions of unidimensionality, local dependence, and monotonicity were assessed.

Item analysis was conducted to estimate internal consistency reliability (Cronbach’s alpha, McDonald’s Omega, and split-half reliability [odd-even]). In addition, the floor and ceiling effect, mean inter-item correlation, corrected item-total correlation, standard error of measurement, Ferguson’s delta, IRT reliability, and Rho coefficient were calculated. Convergent validity was examined using Pearson’s correlation analysis to estimate the correlation of the SAVE-6 with the GAD-7 and PHQ-9. Two-independent sample *t*-tests were performed to examine the mean differences in SAVE-6 scores between high school students with (PHQ-9 ≥ 10) and without depression (PHQ-9 < 10), and between students with (GAD-7 ≥ 10) and without anxiety (GAD-7 < 10). Finally, a receiver operating characteristic (ROC) analysis was conducted to determine the appropriate cut-off score of the SAVE-6 scale in accordance with a mild degree of GAD-7 (≥5). Microsoft Excel 365, IBM SPSS v26, JASP v0.14.1, and Rstudio were used for statistical analysis.

## Results

### Demographic Characteristics

Participants’ demographic characteristics are presented in Table 1. The participants were residents of Seoul (*n* = 55, 18.3%), Pusan (*n* = 9, 3.0%), Daegu (*n* = 15, 5.0%), Daejeon (*n* = 14, 4.7%), Gwangju (*n* = 5, 1.7%), Incheon (*n* = 25, 8.3%), Ulsan (*n* = 8, 2.7%), Gyeonggi Province (*n* = 81, 27.0%), Chungcheong Province (*n* = 19, 6.3%), Jeolla Province (*n* = 16, 5.4%), Gyeongsang Province (*n* = 41, 13.7%), Gangwon Province (*n* = 8, 2.7%), and Jeju Province (*n* = 4, 1.3%).

**TABLE 1:** Clinical characteristics of participants (*N* = 300).

Variables	Mean ± SD, N (%)
**Sex (male)**	150 (50.0%)
**Age, years**	17.0 ± 0.9
**Grade**	
1st grade	100 (33.3%)
2nd grade	100 (33.3%)
3rd grade	100 (33.3%)
**School type**	
High school, general	221 (73.7%)
Special purpose high school	22 (7.3%)
Specialized vocational high school	44 (14.7%)
Autonomous private high school	11 (3.7%)
Others	2 (0.7%)
**Questions on COVID-19**	
Did you experience being quarantined due to infection with COVID-19? (Yes)	68 (22.7%)
Did you experience being infected with COVID-19? (Yes)	5 (1.7%)
Did you get vaccinated? (Yes)	126 (42.0%)
(Among participants who did not get vaccinated: *N* = 174) Do you want to get vaccinated if a vaccine is available? (Yes)	112 (64.4%)
**Psychiatric history**	
Have you experienced or been treated for depression, anxiety, or insomnia? (Yes)	52 (17.3%)
Presently, do you think you are depressed or anxious, or do you need help for your mood state? (Yes)	38 (12.7%)
**Rating scales**	
Stress and Anxiety to Viral Epidemics-6 Items	14.4 ± 5.0
Generalized Anxiety Disorders-7 Items	4.5 ± 5.0
Patients Health Questionnaire-9 Items	7.9 ± 6.6

### Initial Exploratory Factor Analysis

The normality assumption for all six items of the SAVE-6 was checked. We observed that all items were normally distributed according to skewness and kurtosis, which were within the range of ± 2 (Table 2). The KMO measure (0.87) and Bartlett’s test of sphericity (*p* < 0.001) confirmed that the data were suitable for factor analysis. A single factor model of the SAVE-6 was confirmed by the results of a scree plot and EFA with oblimin rotation.

**TABLE 2:** Item properties of the SAVE-6 scale among high school students.

Items	Response scale (%)					Descriptive statistics				CITC	CID	Factor loading (95% CI)
	0	1	2	3	4	M	SD	Skewness	Kurtosis			
Item 1. Are you afraid the virus outbreak will continue indefinitely?	3.3	6.7	21.7	48.0	20.3	2.75	0.96	–0.842	0.657	0.555	0.831	0.609 (0.503, 0.716)
Item 2. Are you afraid your health will worsen because of the virus?	4.3	14.0	21.7	39.7	20.3	2.58	1.09	–0.553	-0.415	0.713	0.801	0.794 (0.684, 0.904)
Item 3. Are you worried that you might get infected?	6.7	12.0	22.7	35.7	23.0	2.56	1.16	–0.586	-0.442	0.740	0.794	0.831 (0.7118, 0.943)
Item 4. Are you more sensitive toward minor physical symptoms than usual?	8.7	18.3	27.7	33.7	11.7	2.21	1.14	–0.303	-0.703	0.604	0.822	0.659 (0.564,0.754)
Item 5. Are you worried that others might avoid you even after the infection risk has been minimized?	23.0	29.7	20.0	19.7	7.7	1.59	1.25	0.330	-0.983	0.510	0.844	0.554 (0.472, 0.636)
Item 6. Do you worry that your family or friends may become infected because of you?	4.0	8.3	22.0	40.3	25.3	2.75	1.05	–0.746	0.105	0.644	0.815	0.709 (0.601, 0.817)

*0 = never, 1 = rarely, 2 = sometimes, 4 = often, 5 = always, M = mean, SD = standard deviation, CITC = corrected item-total correlation, CID = Cronbach’s alpha if item deleted, CI = confidence interval.*

### Confirmatory Factor Analysis

**FIGURE 1:**
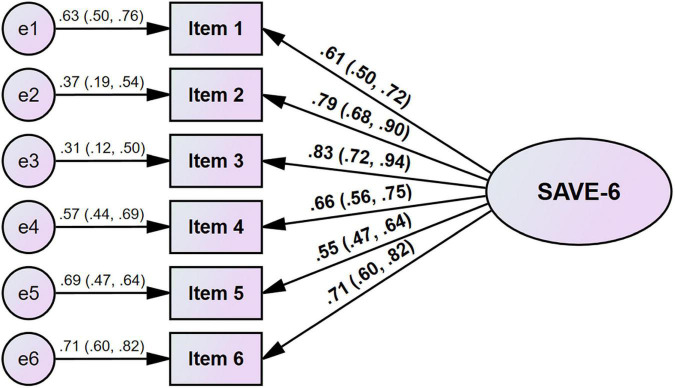
Factor structure of the SAVE-6 among high school students.

The CFA (estimation method = DWLS) showed a good model fit for the SAVE-6 scale among high school students (χ2/df = 0.485, CFI = 1.000, TLI = 1.010, RMSEA = 0.000, and SRMR = 0.029; Table 3). The factor loadings were between 0.554 and 0.794 (Table 2 and Figure 1). The results of the multi-group CFA suggested scalar invariance across sex, grade, and depression (PHQ-9 ≥ 10) (Supplementary Table 1). These results indicated that the SAVE-6 scale can measure the anxiety response of high school students in a similar manner across sex, grade, or depression.

**TABLE 3:** Scale-level psychometric properties of the SAVE-6 among high school students.

Psychometric properties	Scores	Suggested cut-off
Floor effect	1.3	15%
Ceiling effect	2.7	15%
Mean inter-item correlation	0.478	Between 0.15 and 0.50
Cronbach’s alpha	0.844	≥0.7
McDonald’s Omega	0.848	≥0.7
Split-half reliability (odd-even)	0.883	≥0.7
Standard error of measurement	1.979	Smaller than SD (2.51)/2
Ferguson delta	0.978	≥0.9
*Rho* coefficient	0.875	≥0.7
IRT reliability	0.851	≥0.7
**Model fit of confirmatory factor analysis**
χ ^2^ (df, *p* value), χ ^2^/df	4.368 (9, 0.886), 0.485	Non-significant, <5
CFI	1.00	>0.95
TLI	1.01	>0.95
RMSEA [90% CI value] (*p* value)	0.000 [0.000, 0.031] (0.987)	<0.08
SRMR	0.029	<0.08

### Graded Response Model Analysis

The results for the assumptions of the modern test theory showed that, for high school students, the SAVE-6 is strongly unidimensional. An absence of local dependence was evident, as all *p* values (adjusted for false discovery rate) of the G^2^ were non-significant. The assumption of monotonicity was also met, as the absence of significant violation values was evident. These results suggested that all the assumptions for applying a modern test theory model were met. Supplementary Table 2 shows the slope parameters of the SAVE-6 for high school students, ranging from 1.367 to 3.373 (mean = 2.151). Items 1, 4, and 5 had a high slope parameter, and Items 2, 3, and 6 had a very high slope parameter. These high and very high slope parameters demonstrated the efficiency of items in providing reasonable information about the latent trait assessed by the SAVE-6 for high school students. The threshold parameters (Supplementary Table 2) suggested that Item 5 is the most difficult of all items, followed by Item 4. Item 5 had only one negative coefficient and the other three coefficients were positive. This suggested that an above average level of latent trait or theta is required to endorse Likert-type response options from “sometimes” to “always.” For Items 1, 2, 3, and 6, an above average level of latent trait or theta was required to endorse the Likert-type response option “always.” The scale information curve (Supplementary Figures 1, 2) presented an improved understanding of the information provided by the SAVE-6 for high school students. Based on the curve, this scale contributed further details about high school students between the −1.75 and −0.15 θ levels. There were two peaks in the curve, which might be due to the polytomous nature of the data.

### Reliability, Evidence Based on Relationships With Other Variables, and Cut-Off Value

The SAVE-6 scale showed good reliability (Cronbach’s alpha = 0.844 and McDonald’s Omega = 0.848, split-half reliability [odd-even] = 0.883). When an item was dropped, the Cronbach’s alpha ranged from 0.794 to 0.844. The mean inter-item correlation (0.478) was between the recommended range (0.15–0.50). The scale also demonstrated good IRT reliability (0.851) and Rho coefficient (0.875) and had good discrimination power (Ferguson’s delta = 0.978). The total SAVE-6 score was significantly correlated with the scores of the GAD-7 [*r* = 0.387 (95% CI, 0.287, 0.479), *p* < 0.001] and PHQ-9 [*r* = 0.161 (95% CI, 0.048, 0.269), *p* < 0.001]. The SAVE-6 score was significantly higher among students having depressive symptoms [PHQ-9 ≥ 10, *t*(298) = 2.300, *p* = 0.022] or anxiety [GAD-7 ≥ 10, *t*(298) = 4.478, *p* < 0.001]. In addition, the SAVE-6 score was significantly higher among girls than boys [*t*(298) = 3.234, *p* < 0.001] but did not differ significantly among students according to their vaccination status [*t*(298) = 0.801, *p* = 0.424].

The ROC analysis, conducted to determine the appropriate cut-off score for the SAVE-6, showed that a score of 15 was an appropriate cut-off point in accordance with a mild degree of anxiety assessed through the GAD-7 (≥5; area under the curve [AUC] = 0.694, sensitivity = 0.71, specificity = 0.58).

## Discussion

In this study, we aimed to assess the psychometric properties of the SAVE-6 among high school students, who were faced with a stressful situation during the COVID-19 pandemic. The current study confirmed the reliability and validity of the SAVE-6 among high school students, similar to previous studies that evaluated its factor structure for other populations (7, 9–12). In addition, we observed that the SAVE-6 could measure anxiety responses for boys or girls, according to different grades, as well as the incidence of depression among them.

The SAVE-6 scale was derived from the SAVE-9 scale, which was developed to measure healthcare workers’ work-related stress and anxiety response to a viral epidemic. The SAVE-6 was derived from factor I of the SAVE-9 scale (anxiety about the viral epidemic), which was applied to the general population during the COVID-19 pandemic. In this study, we added evidence of the applicability of the SAVE-6 scale not just for the adult population (≥18 years) (7, 9–12) but also for high school students. Given the mental health difficulties that high school students are experiencing during the COVID-19 pandemic, the SAVE-6 can serve as a valid and reliable screening tool to assess the stress and anxiety symptoms of those at risk. Further research is required to verify the psychometric properties of the SAVE-6 in younger students.

The SAVE-6 score was significantly higher among high school students who were reported having depression or anxiety compared to those without depression or anxiety. This result reflects that the SAVE-6 scale can help assess high school students’ anxiety response to viral epidemics efficiently. In this study, we observed that the SAVE-6 score was significantly higher among girls than boys. In previous studies, the SAVE-6 score was found to be significantly higher among females than males (7, 9, 12). This female preponderance concerning viral anxiety needs to be considered when developing viral anxiety scales.

The factor loading of Item 5 was slightly low, but acceptable. Students’ adaptation to the COVID-19 pandemic might be a reason for these lower factor loadings. The survey was conducted in October 2021, when 72% of nationals in South Korea were vaccinated (15). At the time, the government also envisioned the possibility of “living with Corona,” which means treating COVID-19 like the seasonal influenza, and eased the social distancing guidelines (27). In addition, in this study, only 42.0% of participants reported being vaccinated. The Korean government began the vaccination of high school students in mid-July 2021 (28), which may have influenced the results.

The cut-off score of the SAVE-6 among high school students was determined as 15 for this sample. Previously, we explored the cut-off point of the SAVE-6 scale, which was reported as 12–16, in accordance with a mild degree of generalized anxiety (GAD-7 ≥ 5). In studies conducted among the general population (7) and medical students (11), 15 was determined as the cut-off score, similar to this research. While the cut-off score can be influenced by cultural or group differences, we considered a score of 15 on the SAVE-6 scale as the optimal cut-off score for viral anxiety, which accords with the score for mild anxiety in the GAD-7. The low specificity (0.58) of the 15 points on the SAVE-6 scale must be addressed, although the sensitivity (0.71) was sufficient. However, it should be noted that we tried to validate a rating scale to measure the anxiety response specific to this viral epidemic. The GAD-7, based on which we defined the cut-off score for the SAVE-6, does not assess anxiety specific to this viral epidemic. Thus, we do not have a “gold standard” for a viral anxiety measurement tool. Therefore, the cut-off score of the SAVE-6 is not suited for specificity but aims for a similar level of the SAVE-6 scale in accordance with a popularly used rating scale.

This study had several limitations. First, it was conducted via an online survey system rather than through a face-to-face, structured interview format. This may lead to bias, as the enrolled participants were registered as panelists in the survey company system. Furthermore, the anonymous online survey may have affected the reliability of responses. However, we were able to include participants from all areas of South Korea via this system without fear of spreading the virus, a risk factor in face-to-face interviews. Second, a small number of confirmed cases (*n* = 5, 1.7%) and a relatively high proportion of participants in this study receiving vaccination may have influenced the results. Until now, we did not report the validation results among participants who were vaccinated. In addition, 53.7% of participants in our previous study (9) reported being infected. This discrepancy may influence the differences between various studies in terms of validating the SAVE-6. Third, the status of the pandemic and educational environment such as the school or teaching system, availability of remote learning methods, or cultural differences related to education might influence students’ anxiety level.

In conclusion, we observed that the SAVE-6 has good reliability and validity, and it can be applied to assess high school students’ anxiety response to the COVID-19 pandemic. During this pandemic era, we hope that the SAVE-6 scale can be applied to measure the viral anxiety of high school students who are in stressful situations and need psychological support.

## Data Availability Statement

The raw data supporting the conclusions of this article will be made available by the authors, without undue reservation.

## Ethics Statement

The studies involving human participants were reviewed and approved by the Institutional Review Board of Asan Medical Center (2021-1361), which waived the requirement to obtain written informed consent. Written informed consent from the participants’ legal guardian/next of kin was not required to participate in this study in accordance with the national legislation and the institutional requirements.

## Author Contributions

SC and ÖFA contributed to conception and design of the study. TL and SC collected the data. OA and SC performed the statistical analysis. TL, OA, SC, and ÖFA wrote the first draft of the manuscript. All authors contributed to manuscript revision, read, and approved the submitted version.

## Conflict of Interest

The authors declare that the research was conducted in the absence of any commercial or financial relationships that could be construed as a potential conflict of interest.

## Publisher’s Note

All claims expressed in this article are solely those of the authors and do not necessarily represent those of their affiliated organizations, or those of the publisher, the editors and the reviewers. Any product that may be evaluated in this article, or claim that may be made by its manufacturer, is not guaranteed or endorsed by the publisher.

## Footnotes

1
www.embrain.com

2
www.save-viralepidemic.net

3
www.phqscreeners.com
