# Supplementary material for: Psychometric Properties of the Stress and Anxiety to Viral Epidemics-6 Items (SAVE-6) Scale for High School Students During the COVID-19 Pandemic
Source: Front Psychiatry. 2022 May 4;13:815339. doi: 10.3389/fpsyt.2022.815339 (PMC9116484; doi:10.3389/fpsyt.2022.815339)
Supplement: Supplementary file 1 [file Data_Sheet_1.docx]

**Supplementary Table 1. Measurement invariance of the SAVE-6 for high school students across sex, grade, and depression**

| **Model** | **χ^2^** | **df** | **Δ χ^2^** | **Δdf** | **p** | **CFI** | **ΔCFI** |
| --- | --- | --- | --- | --- | --- | --- | --- |
| **Sex** | | | | | | | |
| **Configural** | 8.665 | 18 |  |  |  | 1.000 |  |
| **Metric** | 20.121 | 23 | 11.456 | 5 | .043 | 1.000 | .000 |
| **Scalar** | 28.402 | 28 | 8.281 | 5 | .141 | .999 | .001 |
| **Grades** | | | | | | | |
| **Configural** | 8.085 | 27 |  |  |  | 1.000 |  |
| **Metric** | 22.263 | 37 | 14.178 | 10 | .165 | 1.000 | 000 |
| **Scalar** | 25.701 | 47 | 3.438 | 10 | .969 | 1.000 | 000 |
| **PHQ-9 ≥ 10** | | | | | | | |
| **Configural** | 7.065 | 188 |  |  |  | 1.000 |  |
| **Metric** | 10.155 | 23 | 3.09 | 5 | .686 | 1.000 | 000 |
| **Scalar** | 13.299 | 28 | 3.144 | 5 | .678 | 1.000 | 000 |

**Supplementary Table 2. Slope and threshold parameters of the SAVE-6 among high school students**

| **Items** | **Slope parameter (a)** | **Threshold parameter (b)** | | | |
| --- | --- | --- | --- | --- | --- |
|  |  | **b_1_** | **b_2_** | **b_3_** | **b_4_** |
| **Item 1** | 1.600 | -2.756 | -1.811 | -.620 | 1.221 |
| **Item 2** | 2.809 | -2.064 | -1.027 | -.264 | .951 |
| **Item 3** | 3.373 | -1.679 | -.962 | -.235 | .798 |
| **Item 4** | 1.686 | -1.956 | -.826 | .162 | 1.666 |
| **Item 5** | 1.367 | -1.169 | .069 | .894 | 2.281 |
| **Item 6** | 2.071 | -2.310 | -1.464 | -.504 | .835 |

**Supplementary Figure 1. Scale information curve of the SAVE-6 among high school students**


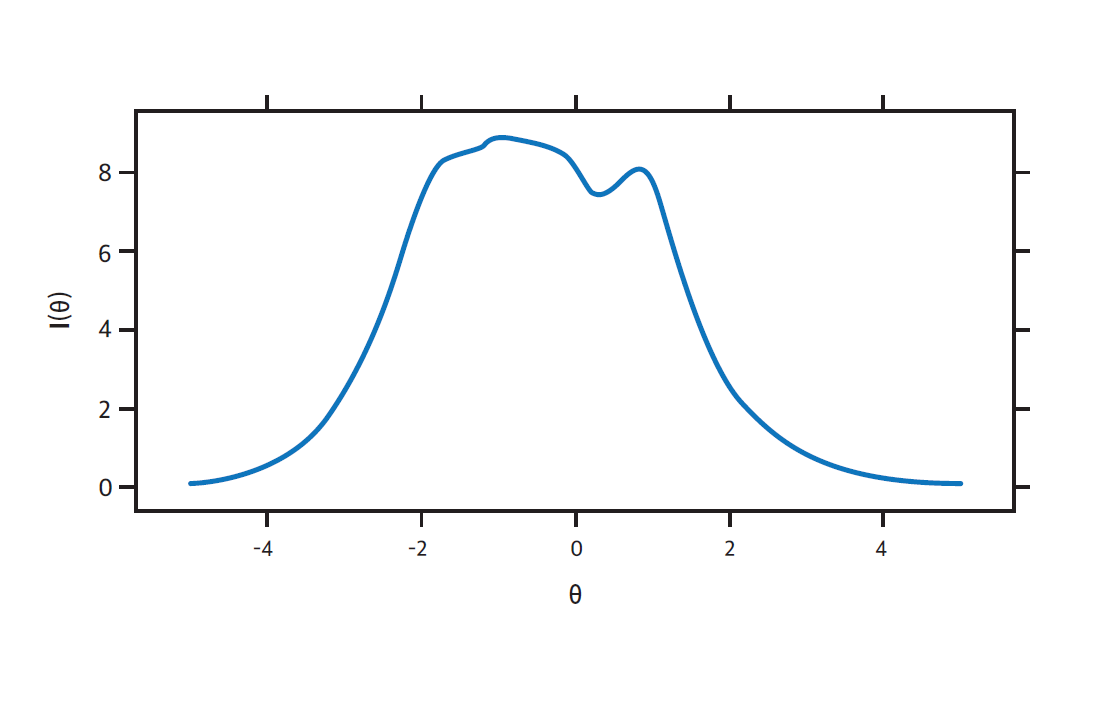


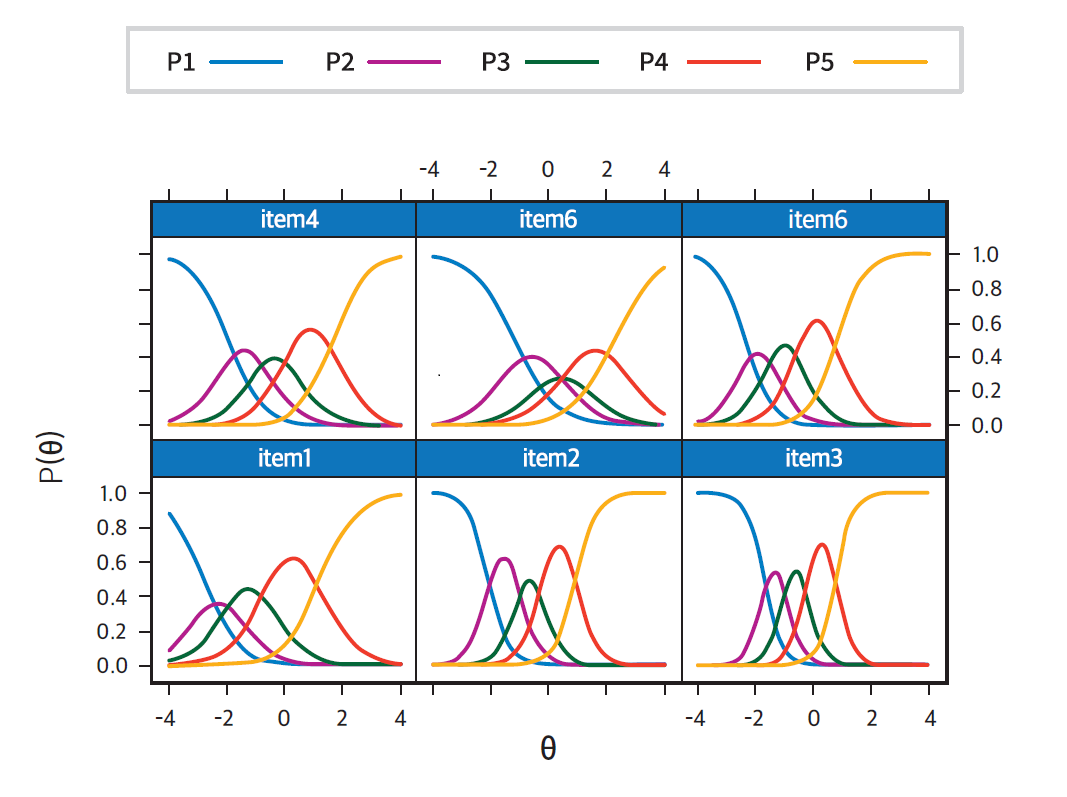


**Supplementary Figure 2. Item trace lines of the SAVE-6 scale among high school students**
